# Transcriptomics and metagenomics of common cutworm (*Spodoptera litura*) and fall armyworm (*Spodoptera frugiperda*) demonstrate differences in detoxification and development

**DOI:** 10.1186/s12864-022-08613-6

**Published:** 2022-05-20

**Authors:** Ruixiang Tang, Fangyuan Liu, Yue Lan, Jiao Wang, Lei Wang, Jing Li, Xu Liu, Zhenxin Fan, Tao Guo, Bisong Yue

**Affiliations:** 1 Key Laboratory of Bio-Resources and Eco-Environment (Ministry of Education), College of Life Sciences, Sichuan University, Chengdu, 610065 Sichuan China; 2 Sichuan Key Laboratory of Conservation Biology On Endangered Wildlife, College of Life Sciences, Sichuan University, Chengdu, 610064 China; 3 Institute of Plant Protection, Sichuan Academy of Agricultural Sciences, Chengdu, 610066 Sichuan China; 4 Department of Obstetrics and Gynecology, West China Second University Hospital, Sichuan University, Chengdu, 610041 Sichuan China

**Keywords:** *Spodoptera litura*, *Spodoptera frugiperda*, Developmental stages, Transcriptome, Metagenomics

## Abstract

**Background:**

*Spodoptera litura* is an important polyphagous pest that causes significant damage to the agricultural sector. We performed RNA-seq of 15 *S. litura* individuals from larval (fifth and sixth instar larvae), chrysalis, and adult developmental stages. We also compared the *S. litura* transcriptome data with *Spodoptera frugiperda* across the same developmental stages, which was sequenced in our previous study.

**Results:**

A total of 101,885 differentially expressed transcripts (DETs) were identified in *S. litura*. Gene Ontology (GO) and Kyoto Encyclopedia of Genes and Genomes (KEGG) enrichment analyses indicated that *S. litura* may undergo active xenobiotic and detoxifying metabolism during its larval and adult stages, which may explain difficulties with current population control measures. We also found that DETs of single-copy orthologous genes between *S. litura* and *S. frugiperda* were involved in basic metabolism and development. However, energy and metabolic processes genes had a higher expression in *S. litura*, whereas nervous and olfactory function genes had a higher expression in *S. frugiperda*. Metagenomics analysis in larval *S. litura* and *S. frugiperda* revealed that microbiota participate in the detoxification and metabolism processes, but the relative abundance of detoxification-related microbiota was more abundant in *S. frugiperda*. Transcriptome results also confirmed the detoxification-related pathway of *S. frugiperda* was more abundant than in *S. litura.*

**Conclusions:**

Significant changes at transcriptional level were identified during the different development stages of *S. litura*. Importantly, we also identified detoxification associated genes and gut microbiota between *S. litura* and *S. frugiperda* at different developmental stages, which will be valuable in revealing possible mechanisms of detoxification and development in these two lepidopterans.

**Supplementary Information:**

The online version contains supplementary material available at 10.1186/s12864-022-08613-6.

## Background

*Spodoptera litura* (Lepidoptera: Noctuidae) is a destructive omnivorous pest with a large host range of plants, and feeds on a wide range of crops [1]. It is widely distributed in tropical and subtropical regions, including in Asia, Africa, North America, and Oceania [2, 3]. *Spodoptera litura* is characterized by a short life cycle consisting of eggs, larval, chrysalises and adults [4]. Eggs are typically laid in batches, and covered with a tuft of abdominal hair to protect them from the natural enemies [5]. The greatest food intake occurs gregariously in the larval stage, where the larvae have sensitive chemosensory systems that are highly selective and essential for searching for food [6, 7]. Therefore, there is an urgent need for effective management strategies to control this pest.

Previous studies primarily evaluated the influence of different host plants on the growth and development of *S. litura* [8–10] and its potential damage to different crops [2]. Given the poor efficacy of various insecticides against *S. litura* [3], researchers have also extensively investigated its resistance to insecticides. Transcriptome analysis of *S. litura* demonstrated that P450s may be involved in the detoxification of fluralaner in vivo [11]. Studies also found that the detoxion-related gene families (P450, GST, C0E, APN and ABC) were massively expanded in *S. litura*, which may be a genetic basis for its high tolerance to pesticides [12]. Despite a large volume of research to understand the ecology and genetics of *S. litura*, the developmental characteristics of *S. litura* and the underlying changes during development that allow resistance to pesticides remains poorly understood. RNA sequencing has been widely used to obtain expression data of different developmental stages of agricultural pests and to explore the key regulated genes related to development that can be targeted for controlling pests. Pairwise comparison of four developmental stages of *Athetis lepigone* found that several differentially expressed genes were related to cuticle and wing formation as well as the growth and development [13]. Transcriptome analysis of *Ostrinia furnacalis* in four developmental stages revealed genes were associated with developmental pathways, cuticularization, wing formation and olfactory recognition [14]. Simon, et al., performed analyses of gene expression patterns during *S. exigua* developmental stages and identified four Spodoptera-specific candidate genes for RNAi-based pest management strategies [15]. Consequently, it is necessary to undertake RNA-seq on *S. litura* to compare the differences in gene expression profiles at different stages to design efficient strategies to control this economic pest.

The fall amyworm (*S. frugiperda*) is phylogenetically close to *S. litura* and belongs to the same genus *Spodoptera*. Despite similar biological and morphological characteristics such as high resistance to pesticides, strong reproducibility and migration [16], *S. frugiperda* and *S. litura* differ in their feeding habit, reproductive behavior and damage degree [17]. *Spodoptera frugiperda* prefers gramineous plants, whereas *S. litura* prefer dicotyledons. Another difference is that *S. frugiperda* has more powerful locomotion than *S. litura*. In addition, *S. frugiperda* shows higher mating frequency than *S. litura*. The genetic divergence between the two pests was demonstrated by comparative genomics [16], which found genes associated with sensory perception, such as to taste stimulus, and the nervous system were significantly enriched in *S. frugiperda.* However, the enriched biological processes in *S. litura* were mainly in development, reproduction, and metabolism. Furthermore, our team studied the gene expression differences between developmental stages of *S. frugiperda* [18] and found that *S. frugiperda* experienced active metabolism, detoxifying and xenobiotic metabolism throughout its life, especially in the larval stage. Considering the observed differences in feeding habit and reproductive behavior between *S. frugiperda* and *S. litura*, it is possible that there are gene expression differences between them even during the same development stage. Therefore, comparative transcriptomics between the two pests at different development stages will be helpful to better understand their genetic differences.

**Fig. 1:**
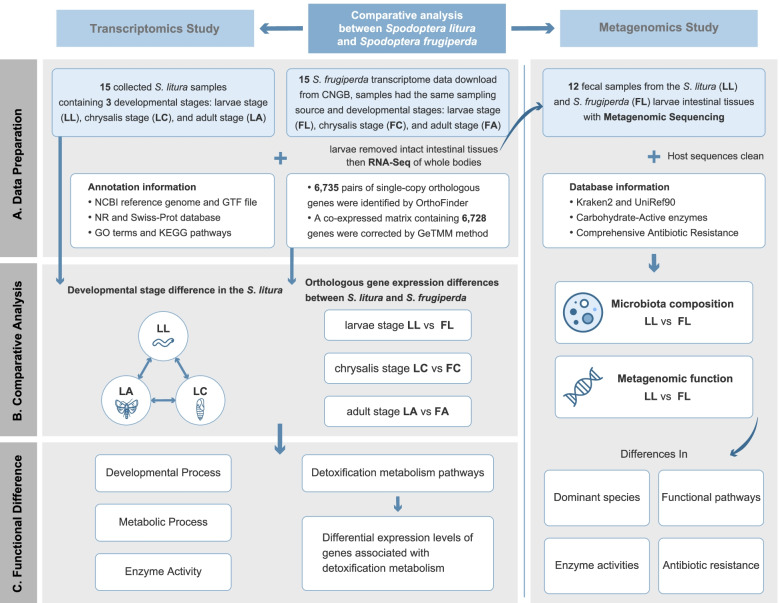
Data processing and comparative analysis workflow of this project. Schematic pipeline illustrating the workflow of transcriptomic and metagenomic analysis between *S. litura* and *S. frugiperda*, as well as some differences in function we found

Another aspect that could increase these pests invasive and destructive capabilities is how their gut microbes contribute to food digestion and protection against pathogens. Analysis of the metagenome of *S. litura* larvae revealed that microbiota played a major role in digestion, detoxification, and nutrient supply [19, 20]. While *S. frugiperda* gut bacteria participated in modulate plant defense responses [21]. However, no comparative study of gut microbial differences between *S. litura* and *S. frugiperda* exists. Therefore, we aim to investigate the differences of gut microflora composition and functional annotation of *S. litura* and *S. frugiperda* using metagenomics. In this study, 15 *S. litura* individuals, including larval stage (marked as LL, contained fifth instar larvae and sixth instar larvae, marked as L5 and L6), chrysalis stage (marked as LC) and adult stage (marked as LA, contained female and male, marked as LAF and LAM), were collected and we performed RNA-seq across stages and metagenomics on the larval stage. We identified DETs between development stages and identified metabolic pathways that changed with development. Furthermore, we collected and re-analyzed the data of *S. frugiperda* from Wang et al., consisting of 15 *S. frugiperda* individuals, including larval stage (marked as FL, contained fifth instar larvae and sixth instar larvae, marked as F5 and F6), chrysalis stage (marked as FC) and adult stage (marked as FA, contained female and male, marked as FAF and FAM), that identified detoxifying-related metabolism and basic metabolism differences with development of *S. litura* and *S. frugiperda* [18]. The metagenomics characterized the structure of the gut bacterial communities and found a significant difference between the two species at the genus level, with the bacterial composition and diversity of *S. frugiperda* guts being more diverse than *S. litura*, and the genes related to energy metabolism process and detoxification differed (Fig. 1). This study provided new insights into understanding and utilizing the genetic differences for formulating specific strategies to control these two invasive pests.

## Results

### Transcriptome sequencing and alignment

To explore the transcript expression patterns of *S. litura* at different developmental stages, we collected 15 *S. litura* samples from three developmental stages, larvae, chrysalis, and adults. Transcriptome sequencing of the cDNA library was performed on the Illumina HiSeq 2000 platform. A total of 446,991,085 raw reads and 134.1 Gb data were obtained from all samples. After quality control of the transcripts, 436,543,284 (97.66%) clean reads were obtained. We then mapped the clean reads to the reference genome and the results showed that the mapping rate was 81.31% ~ 94.96% (Supplementary Table S1).

### Transcript expression analysis

We found that 23,020 transcripts were expressed, which accounted for 88.96% of the known transcripts. Next, DEseq2 was used to standardize the raw expression matrix and then perform the differential transcripts analysis between groups. The principal component analysis (PCA) demonstrated that the larvae, chrysalis, and adult groups of *S. litura* were clearly separated. At the same time, there was also a clear separation between the female and male adults (Figure S1).

### Developmental differences in *S. litura*

**Fig. 2:**
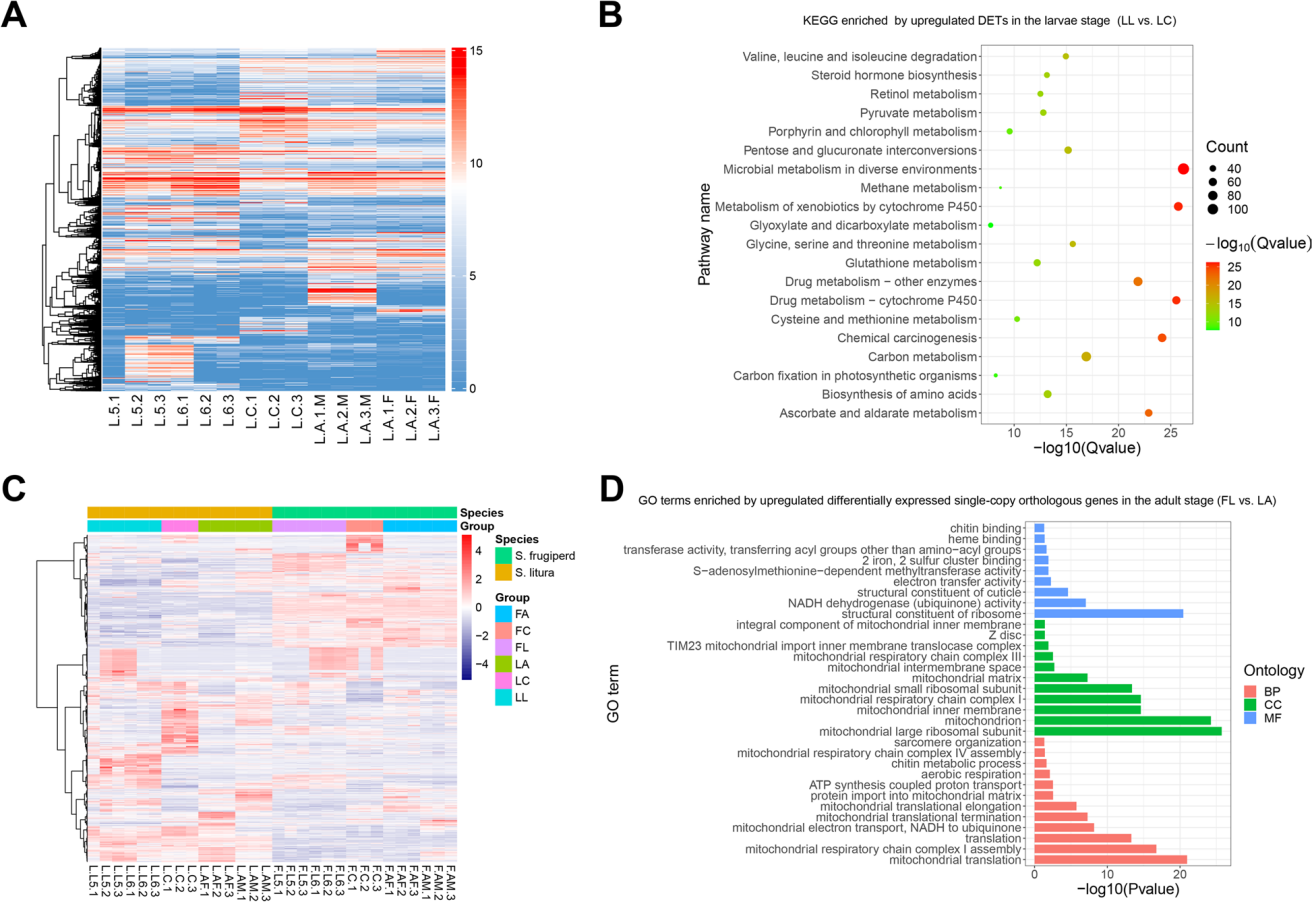
The expression and functional enrichment of DETs in S. litura and between S. litura and S. frugiperda. **(A)** The heatmap of expression of all DETs identified by differentially expressed analysis in *S. litura*. The expression of DETs was normalized using rlog function. Red color represented most abundance and blue color represented less abundance. **(B)** The KEGG pathways enriched by upregulated DETs in the larvae stage when compared to the chrysalis stage (LL vs. LC). **(C)** The heatmap of expression of all differentially expressed single-copy orthologous genes between *S. litura* and *S. frugiperda* identified by differentially expressed analysis. Red color represented most abundance and blue color represented less abundance. **(D)** The top20 significant GO terms enriched by upregulated differentially expressed single-copy orthologous genes between *S. litura* and *S. frugiperda* in the adult stage (FL vs. LA). LL vs. LC, larvae group versus chrysalis group of *S. litura*; FL vs. LA, larvae group versus adult group of *S. frugiperda*; DETs, differentially expressed transcripts; GO, gene ontology; Count means the number of genes enriched in pathway; Q-value is the value of P-value after multiple hypothesis testing and correction

Through cross-comparison of DETs at three developmental stages, a total of 2,703 downregulated DETs and 4,270 upregulated DETs were identified in the larval group (LL) when compared to the chrysalis group (LC). There were 3,145 downregulated DETs and 1,847 upregulated DETs in the LC group when compared to the adult group (LA). In addition, 2,979 downregulated DETs and 3,629 upregulated DETs were identified in the LL group when compared to LA (Fig. 2A). The Venn diagram illustrated the number of common and unique DETs between pairwise comparison of groups (Figure S2).

In order to understand the biological role of DETs, GO and KEGG function enrichment analysis was performed. The upregulated DETs in the LL group (i.e., LL vs. LC) were mainly enriched in biological process (BP) and molecular function (MF) GO terms, such as lipid catabolic process (GO:0,016,042), carbohydrate metabolic process (GO:0,005,975), glutathione metabolic process (GO:0,006,749) and trehalose transport (GO:0,015,771) in BP terms, which all related to basic metabolism processes. Many enzyme-related MF terms were enriched by these DETs, such as glutathione transferase activity (GO:0,004,364) and oxidoreductase activity (GO:0,016,491) (Supplementary Table S2). In KEGG enriched metabolic pathways, detoxification metabolism-related KEGG pathways were also enriched such as drug metabolism—cytochrome P450 (ko00982) and drug metabolism—other enzymes (ko00983) (Fig. 2B and Supplementary Table S2). Downregulated DETs in the LL group were mainly enriched in the development BP terms, such as locomotion (GO:0,040,011) and muscle contraction (GO:0,006,936). In MP terms, many cell activity processes were enriched, such as structural constituent of muscle (GO:0,008,307), actin filament binding (GO:0,051,015) and actin-dependent ATPase activity (GO:0,030,898) (Supplementary Table S2). Moreover, many signaling KEGG pathways, such as ErbB signaling pathway (ko04012), Rap1 signaling pathway (ko04015) and Hippo signaling pathway (ko04390) were enriched (Supplementary Table S2).

In the LC group (i.e., LC vs. LA), upregulated DETs were mainly enriched in development BP terms, including muscle organ development (GO:0,007,517), tissue development (GO:0,009,888) and chitin metabolic process (GO:0,006,030). In MP terms, many DETs were associated with chitin metabolism, such as chitin binding (GO:0,008,061), structural constituent of pupal chitin-based cuticle (GO:0,008,011) and chitinase activity (GO:0,004,568) (Supplementary Table S3). Some basic metabolisms in the KEGG pathways were enriched, such as citrate cycle (TCA cycle) (ko00020) and oxidative phosphorylation (ko00190) (Supplementary Table S3). The majority of downregulated DETs were enriched in energy-related and behavior BP terms, including trehalose transport (GO:0,015,771), disaccharide transport (GO:0,015,766) and chorion-containing eggshell formation (GO:0,007,304). MP terms that were associated with enzyme activity were abundantly enriched, such as serine-type endopeptidase activity (GO:0,004,252) and serine hydrolase activity (GO:0,017,171) (Supplementary Table S3). For KEGG pathways analysis, many detoxification metabolism pathways were enriched, including metabolism of xenobiotics by cytochrome P450 (ko00980), drug metabolism—cytochrome P450 (ko00982) and drug metabolism—other enzymes (ko00983) (Supplementary Table S3).

Upregulated DETs in the LL group (i.e., LL vs. LA) were mainly enriched in developmental BP terms, such as axoneme assembly (GO:0,035,082) and cytoplasmic translation (GO:0,002,181). In MF terms, many terms that were associated with enzyme activity were enriched, including glutathione transferase activity (GO:0,004,364) and serine-type endopeptidase activity (GO:0,004,252) (Supplementary Table S4). In the KEGG analysis, many pathways related to energy metabolism and detoxification metabolism were enriched, such as citrate cycle (TCA cycle) (ko00020) and metabolism of xenobiotics by cytochrome P450 (ko00980) (Supplementary Table S4). Downregulated DETs in the LL group were mainly enriched in BP terms that were associated with the nervous system, including axon guidance (GO:0,007,411), neuron differentiation (GO:0,030,182) and nervous system development (GO:0,007,399) (Supplementary Table S4). There was only one KEGG pathway, lysosome (ko04142), which was enriched by these downregulated DETs (Supplementary Table S4).

In general, we detected many BP terms that were associated with development and basic metabolism and MF terms related to enzyme activity in these comparisons. We also found many terms were involved in chitin metabolism. Furthermore, several KEGG pathways related to detoxification were found.

### Gene expression differences between *S. litura *and *S. frugiperda*

In order to analyze the gene expression differences between *S. litura* and *S. frugiperda* at the same age stage, we performed DETs analysis on the shared single-copy orthologous genes. The orthologous genes of two species were selected by the OrthoFinder, and 6,735 pairs of single-copy orthologs were identified after stringent screening. Among them, 6,728 were co-expressed by *S. litura* and *S. frugiperda*. Gene expression between the two species can be quantified and compared after the length correction of corresponding gene CDS region. After that, we compared differential expressions of these single-copy orthologous genes within developmental stages between the two species. We identified 454 downregulated DETs and 440 upregulated DETs in the LL group when compared to the larval group of *S. frugiperda* (hereafter FL, consisting of 5th instar larvae and 6th instar larvae). A total of 493 downregulated DETs and 589 upregulated DETs were identified in the LC group when compared to the chrysalis group of *S. frugiperda* (i.e., FC). In addition, 658 downregulated DETs and 611 upregulated DETs were identified in the LA group when compared to the adult group of *S. frugiperda* (i.e., FA). A heatmap was generated using DETs of single-copy orthologous genes between *S. litura* and *S. frugiperda* across the three developmental stages (Fig. 2C).

We further performed GO and KEGG enrichment analysis of DETs of single-copy orthologous genes. The results showed that upregulated DETs in the LL group (i.e., LL vs. FL) were only enriched in one MF term, structural constituent of cuticle (GO:0,042,302). There was no significant KEGG pathway enriched in upregulated DETs in the LL group. The GO analysis showed that downregulated DETs in the LL group were enriched in one BP term, olfactory learning (GO:0,008,355) and one CC term, synapse (GO:0,045,202), and they were associated with sensory and nervous system development. There were four KEGG pathways enriched by these DETs, Rap1 signaling pathway (ko04015), axon regeneration (ko04361), adherens junction (ko04520) and tight junction (ko04530) (Supplementary Table S5).

The GO analysis showed that upregulated DETs in the LC group (i.e., LC vs. FC) were mainly enriched in, for example, extracellular space (GO:0,005,615), extracellular region (GO:0,005,576) and serine-type endopeptidase activity (GO:0,004,252). There was only one enriched KEGG pathway, fanconi anemia pathway (ko03460). Downregulated DETs in the LC group were enriched in nervous system developmental related CC terms, such as neuromuscular junction (GO:0,031,594). The nervous development and cell activity KEGG pathways that were associated with these downregulated DETs included the axon regeneration (ko04361) and tight junction (ko04530) (Supplementary Table S6).

Lastly, the GO annotation analysis of upregulated DETs in the LA group (i.e., LA vs. FA) were mainly enriched in energy related BP terms such as mitochondrial translation (GO:0,032,543), ATP synthesis coupled proton transport (GO:0,015,986) and aerobic respiration (GO:0,009,060), as well as binding-related MF terms, including chitin binding (GO:0,008,061) and heme binding (GO:0,020,037) (Fig. 2D). The metabolism-related KEGG pathways associated with these DETs included citrate cycle (TCA cycle) (ko00020), oxidative phosphorylation (ko00190) and 2-oxocarboxylic acid metabolism (ko01210) (Supplementary Table S7). The GO and KEGG enrichment analysis showed that there was no significant pathway enriched of downregulated DETs in the LA group.

In summary, the GO and KEGG enrichment analysis showed that the DETs of single-copy orthologous genes between *S. litura* and *S. frugiperda* were involved in basic metabolism and development. At the larval stage, downregulated DETs in *S. litura* were enriched in a few pathways related to nervous system development and cell activity. At the chrysalis stage, there were more pathways associated with development in *S. frugiperda* than in *S. litura*. At the adult stage, the upregulated DETs of single-copy orthologous genes in *S. litura* were enriched with more terms and pathways related to energy metabolism than *S. frugiperda*.

**Fig. 3:**
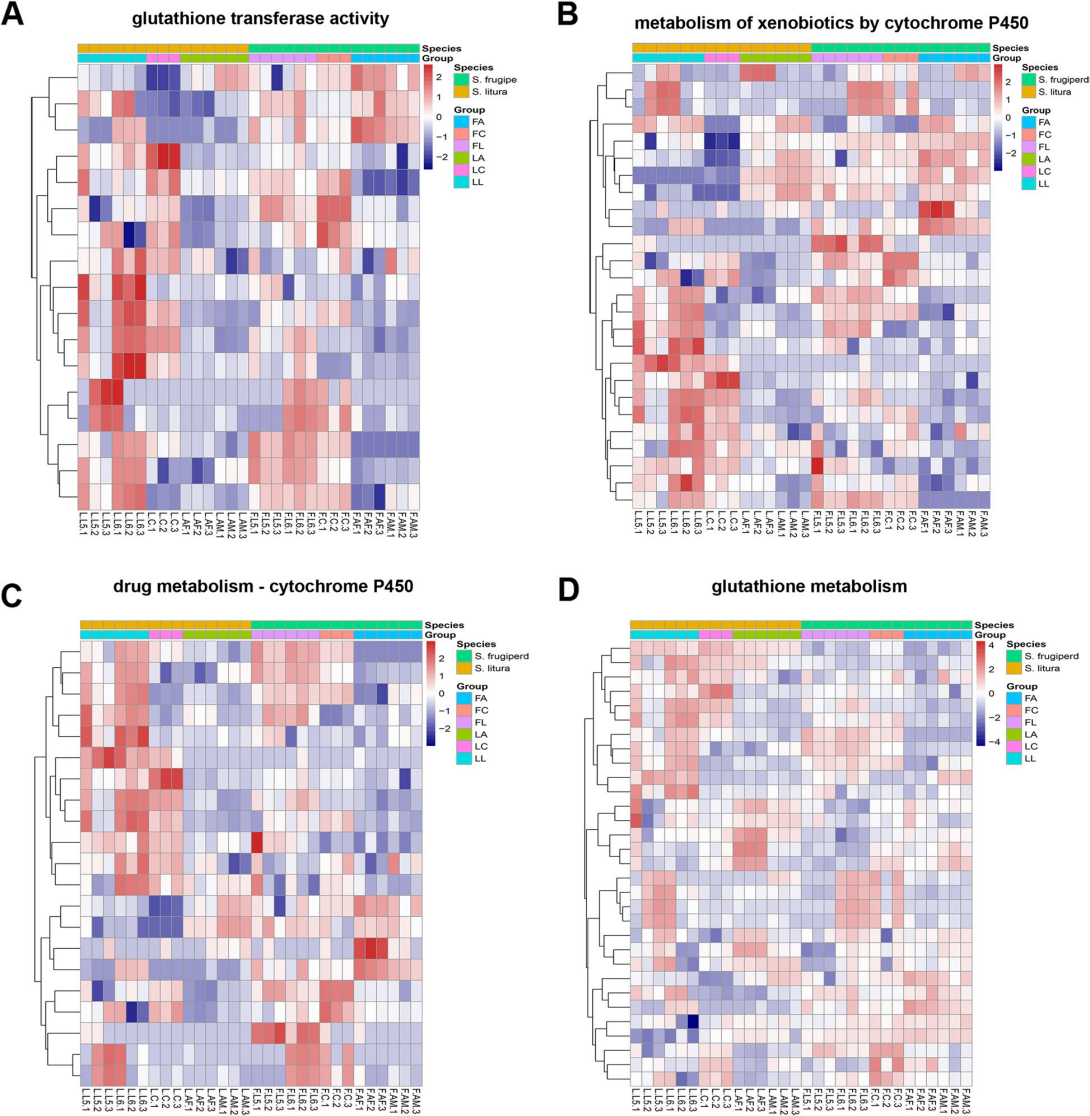
The heatmap of expression of single-copy orthologous genes involved in detoxification-related pathways in S. litura and S. frugiperda. Red color represented most abundance and blue color represented less abundance. **(A)** The heatmap of the expressions of genes which involved in glutathione transferase activity. **(B)** The heatmap of the expressions of genes which involved in metabolism of xenobiotics by cytochrome P450. **(C)** The heatmap of the expressions of genes which involved in drug metabolism—cytochrome P450. **(D)** The heatmap of the expressions of genes which involved in glutathione metabolism

Next, we focused on the detoxification genes. In the past studies, it was found that some enzymes in *S. litura*, such as cytochrome P450, carboxylesterase and glutathione-s-transferase, which would contribute to the host's adaptability of detoxification [22]. In order to further explore the differences of detoxification ability between *S. litura* and *S. frugiperda*, we compared the expression levels of their homologous genes involved in detoxification pathways related to P450, GST, and carboxylesterase. The heatmap showed the expression of homologous genes involved in detoxification pathways shared by both species at different stages, including glutathione transferase activity (Fig. 3A), metabolism of xenobiotics by cytochrome P450 (Fig. 3B), drug metabolism—cytochrome P450 (Fig. 3C) and glutathione metabolism (Fig. 3D) (Supplementary Table S13). The results showed that the gene expression levels involved in detoxification pathways were different in the three stage groups of the same species. For example, the expression of genes involved in the glutathione transferase activity pathway was significantly higher in the larval stage than other stages in *S. litura* (Fig. 3D). In addition, the gene expression levels involved in detoxification pathways were also different between species within stages. For example, gene expression of xenobiotic metabolism by the cytochrome P450 pathway was highest at the larval stage in *S. litura*, while the expression of genes involved in the same pathway was high at all three stages in *S. frugiperda*. These results clearly indicated that the expression patterns of detoxification related genes during development may differ between the two lepidoptera (Fig. 3D).

### Comparative microbiota composition of *S. litura* and *S. frugiperda*

**Fig. 4:**
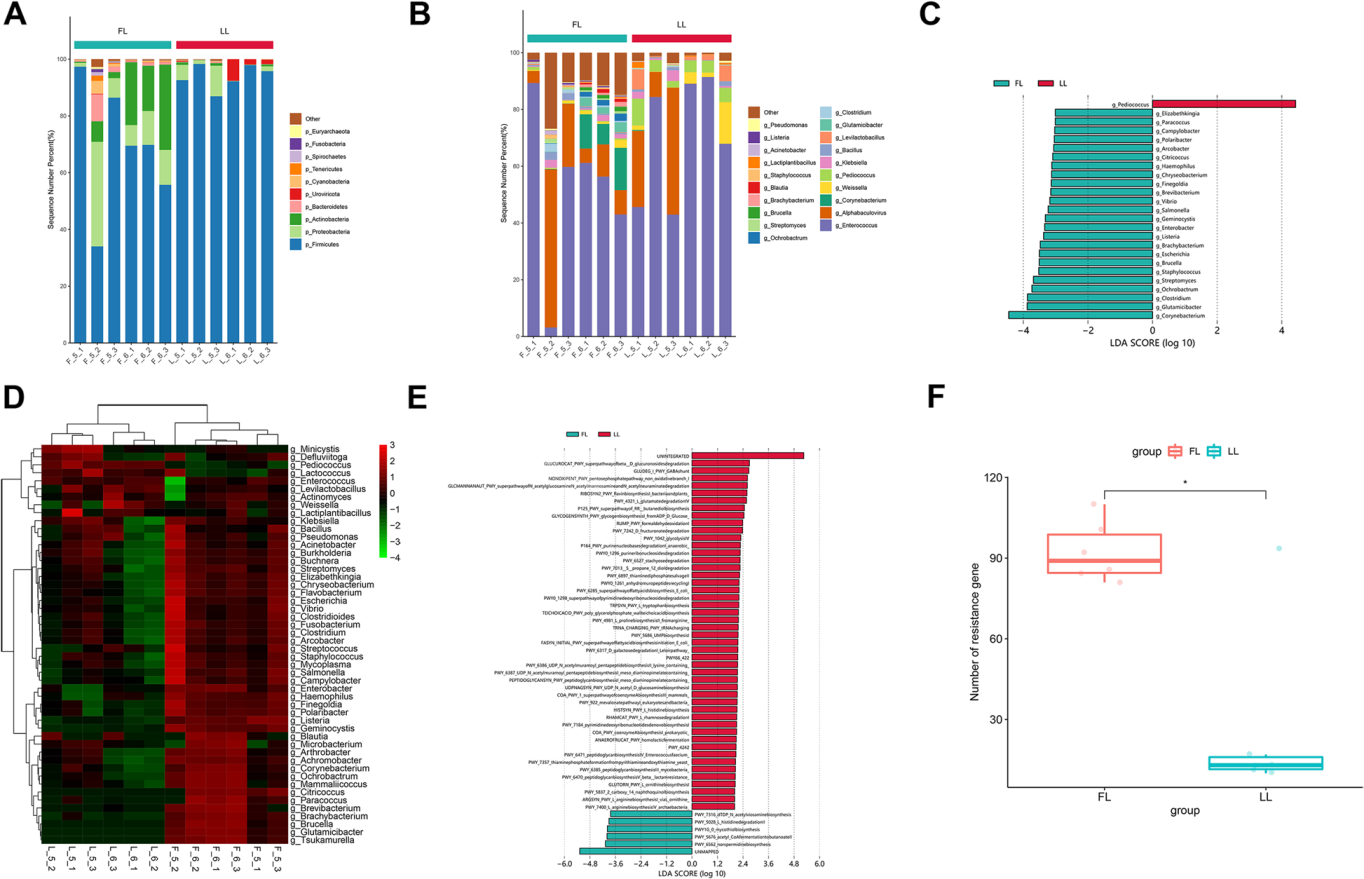
Diversity and function of bacterial communities of S. litura and S. frugiperda. **(A)** Relative abundance of microbial community in all samples at phylum level. **(B)** Relative abundance of microbial community in all samples at genera level. **(C)** Gut microbiota bacterial comparisons between *S. litura* and *S. frugiperda* groups analyzed by LEfSe (LDA > 3, *P* < 0.05) at the genera level. **(D)** Heatmap of the top 50 most abundant genera in bacterial communities of the two species in the 9 samples. Red color represented most abundance and green color represented less abundance. **(E)** LDA effect size (LEfSe) analysis of the function of the unstratified pathways between two groups. **(F)** The boxplot of ARGs annotations number between two groups. ARGs, antibiotic resistance genes

Metagenome sequencing analysis of the larval (fifth instar larvae and sixth instar larvae) stage of *S. litura* and *S. frugiperda* obtained 474,122,459 and 479,748,778 valid reads from six samples each of *S. litura* and *S. frugiperda*, respectively (Supplementary Table S8). A total of 27 phyla, 49 classes, 114 orders, 152 families, 750 genera and 2,047 species were identified in *S. litura*, while 26 phyla, 52 classes,124 orders, 167 families, 881 genera and 2,631 species were found in *S. frugiperda* samples. Firmicutes, Proteobacteria and Actinobacteria were the dominant phyla in all samples. The proportion of Firmicutes was higher in *S. litura* compared to *S. frugiperda* (95.88% and 93.00%), whereas the relative percentage of Proteobacteria composition was higher in *S. frugiperda* (1.30% and 3.00%; Fig. 4A). At the genus level, *Enterococcus*, *Alphabaculovirus* and *Corynebacterium* were the dominant genera in all samples. *Enterococcus*, *Pediococcus* and *Weissella* were the dominant genera of *S. litura*, while *Enterococcus*, *Corynebacterium* and *Bacillus* were the most abundant genera of *S. frugiperda* (Fig. 4B). Linear discriminant analysis effect size was performed to identify differences in gut bacteria at genus level between *S. litura* and *S. frugiperda* at the larval stage. The relative abundance of *Pediococcus* was significantly higher in *S. litura* than in *S. frugiperda*, however, the relative abundance of several genera, such as *Corynebacterium*, *Clostridium* and *Glutamicibacter* were significantly higher in *S. frugiperda* compared to *S. litura* (LDA > 3, *p* < 0.05; Fig. 4C). A heatmap that was constructed based on the abundance of the top 50 genera of each sample (Fig. 4D) also showed significant differences at the genus level.

### Metagenomic functional analysis

To analyze the functional relationships of the composition of *S. litura* and *S. frugiperda* metagenomes, genes were predicted from the CAZy, Humann3 and CARD databases. A total of 302 annotated genes from six families, consisting of glycoside hydrolases (GHs), glycosyltransferases (GTs), polysaccharide lyases (PLs), carbohydrate esterases (CEs), auxiliary activities (AAs), and carbohydrate binding modules (CBMs), were detected in the gut microbiota based on the Carbohydrate-Active Enzymes (CAZy) database (http://www.cazy.org). In *S. litura*, we obtained 170 GH subfamilies, 74 GT subfamilies, 48 CBM subfamilies, 17 CE subfamilies, 24 PL subfamilies, and 16 AA subfamilies. However, *S. frugiperda* samples identified 134 GH subfamilies, 64 GT subfamilies, 40 CBM subfamilies, 17 CE subfamilies, 14 PL subfamilies and 11 AA subfamilies. The most abundant CAZyme families detected were represented by subfamilies GH38 and GH85 in GH families. Moreover, LEfse identified GT31, GH13_25 and GT10 as the top three significantly more abundant CAZymes in *S. frugiperda* than in *S. litura*. While GH13_14, GH67 and GH94 were the top three significantly more abundant CAZymes in *S. litura* than *S. frugiperda* (LDA > 2, *P* < 0.05; Figure S3 and Supplementary Table S9).

Metagenomic functional profiling was performed using Humann3 and the pathway abundances were compared using LEfSe with the LDA > 2. In the unstratified pathway analysis, 54 pathways differed between the *S. litura* and *S. frugiperda* groups. Among them, 48 pathways, such as mycothiol biosynthesis, were more abundant in the *S. frugiperda* group. In contrast, six pathways, including the pentose phosphate pathway, were more abundant in the *S. litura* group (Fig. 4E). The relative abundance of all pathways is summarized in Supplementary data, Table S10.

Next, a total of 407 antibiotic resistance genes (AGRs) were detected in the gut microbiomes. We detected that *S. frugiperda* had 330 resistance gene types, while *S. litura* had less, only 117 gene types (Fig. 4F). Of all the AGRs, the relative abundances of AbaF, Trimethoprim, and MexD were most abundant in *S. frugiperda*, and Trimethoprim, vanRC, and efrA genes were most abundant in *S. litura*. We only found 289 genes unique to *S. frugiperda* and 76 genes were unique to *S. litura* (Supplementary Table S11). We then matched each resistance gene type to its corresponding antibiotic, and found that the number of cephalosporin and tmacrolide antibiotics were the most in all samples.

## Discussion

*Spodoptera litura* is an important polyphagous agricultural pest, which is difficult to eliminate because it has evolved resistance to many insecticides [23]. Development stage transcriptomes of *S. litura* showed evidence of adaptive evolution of cuticular protein genes. These genes have been sequenced and characterized and results suggested that various cuticular protein groups produce cuticle layers with different physical properties at different stages [24, 25]. However, a few studies have focused on *S. litura* developmental patterns. In the present study, the transcriptome analyses of *S. litura* at three developmental stages were conducted and identified 10,184 DETs through pairwise comparisons (Supplementary Table S12). The most abundant DETs (6,973) were identified between the LL and LC groups, which may be due to physiological processes such as metamorphosis and molting, from larval to chrysalis stage. Meanwhile, we found that most DETs were related to basic metabolism, detoxification, and development. The transcriptome comparative analysis of these two polyphagous species, *S. litura* and *S. frugiperda*, identified that upregulated DETs of single-copy orthologous genes in *S. frugiperda* were enriched in the nervous system and olfactory functions, while those of *S. litura* were enriched in energy metabolism.

*Spodoptera litura* exhibits the typical developmental characteristics at different growth stages. In the larval stage, we discovered that genes involved in the citric acid cycle and glycolysis, related to energy metabolic processes were upregulated (Supplementary Table S2 and S4). These active metabolic processes were conducive to allow larvae to accumulate energy for rapid growth and future metamorphosis and reproduction. The metamorphosis of insects is a complex process accompanied by dynamically morphological and physiological changes [26]. The energy demand decreases sharply after pupation and remains at a low level until the adult stage [25], and could explain why our analysis of the larval stage had the most abundant energy metabolism function. In the chrysalis stage, the GO terms of organ and nervous system development were enriched in the upregulated transcripts compared to larvae and adults (Supplementary Table S2 and S3). Through GO enrichment analysis, we found that the biological functions related to locomotion and actin filament binding were downregulated in the larval stage (LL vs. LC), in consideration of the different locomotive activities of larvae compare to chrysalis, which seems counterintuitive. But these up-regulated genes in chrysalis were more related to muscle composition, muscle cell development, actin silk assembly and actin cytoskeleton organization development, etc., which reflected the large amount of protein synthesis and muscle tissue development in chrysalis, paving the way for the motor ability of adults. Since muscle activity and movement were high-level go terms, they included these genes related to muscle tissue development and were therefore significantly enriched. It was the developmental characteristics, did not mean that the chrysalis had stronger motor ability than the larva. And the chrysalis stage also experiences active organ development. Wang et al., also found that basic metabolism and organ morphogenesis were rather active in the *S. frugiperda* chrysalis stage [18]. Several transcripts involved in chitin metabolism were enhanced significantly in the chrysalis stage. Chitin is involved in the formation of the epidermis, midgut and the peritrophic matrix of an insect and chitin content changes dramatically during development, which is closely related to growth and development [24, 25]. We suggest that the enriched terms of chitin metabolism are associated with metamorphosis, but also benefit the insect by generating tougher cuticles and providing chemical and biological protection in the *S. litura* chrysalis stage. This helps us to develop specific insecticides that target genes related to the metabolism of chitin [27].

In the adult stage, GO terms associated with reproduction were enriched, such as chorion-containing eggshell formation (GO:0,007,304), embryonic pattern specification (GO:0,009,880) and structural constituent of chorion (GO:0,005,213), which are essential to the behaviors like mating, oviposit, and incubation. Fatty acid derivatives are involved in the synthesis of sex pheromones [28]. This was also supported by our GO enrichment results, where the upregulated DETs in the adult stage were enriched in unsaturated fatty acid biosynthetic process and fatty acid biosynthetic process terms.

*Spodoptera litura* have active xenobiotic and detoxifying metabolism in the larval and adult stages. As an important member of the metabolic enzyme superfamily, P450 plays an important role in the metabolism of xenobiotics, such as pesticides and plant secondary metabolites [29]. P450s also are involved in various other processes, such as insect development and reproduction [30]. Glutathione (GSH) and glutathione s-transferase (GST) are important enzymes involved in many processes of pesticide degradation [31]. In our study, the expression level of transcripts associated with P450 and GST in the larval and adult stage was significantly higher than in the chrysalis stage. We found that the larval stage was the most abundant, implying that detoxification ability was the strongest in the larval stage (Supplementary Table S2, S3, and S4). Previous studies have confirmed that fifth-instar *S. litura* larvae begin to respond to the insecticide avermectin via the P450 and glutathione-s-transferase enzymes metabolism mechanism [3]. Furthermore, it was demonstrated that the increased activity of P450 was an important factor in conferring indoxacarb resistance in the fourth-instar larva *S. litura* [32]. The detoxification pathways enriched in adults may be related to their strong migration ability, which is crucial for their survival and adaptation to different hosts. In general, this indicates that insecticide application in the *S. litura* chrysalis stage when there is relatively low transcript expression related to the detoxification pathways may increase insecticide effectiveness. In addition, the plant xenobiotic azadirachtin [33], organophosphate insecticide chlorpyrifos [34] and the newer insecticide indoxacarb [32], may cause the central nervous system dysfunction of pests, thus we considered that these insecticides could be applied in the chrysalis stage when there is more active neurodevelopmental function. The upregulated DETs in the larval stage compared to chrysalis and adults were enriched in many basic metabolic processes (Supplementary Table S2 and S4), such as carbohydrate metabolic process (GO:0,005,975) and glutathione metabolic process (GO:0,006,749), indicating that the larval stage has active metabolism ability. We speculate that this may be related to the unique detoxification ability of the *S. litura* larvae, higher detoxification enzyme activities were found at the larval stage and this may help insecticide metabolism [35]. Larvae is the most important developmental stage that causes damage to host plants and thus the study of the high expression genes related to detoxification function during the larval stage is of great significance for pest control.

In order to compare the differences in gene expression levels between *S. litura* and *S. frugiperda*, we first screened single-copy orthologous genes of the two species and compared their differences. Then, the functional enrichment of the DETs of single-copy orthologous genes was undertaken. The results showed that the upregulated DETs of single-copy orthologous genes in *S. frugiperda* were enriched in nervous and olfactory function related pathways (FL vs. LL and FC vs. LC). This was consistent with genome level studies that showed significant genetic richness in sensory systems, such as taste stimulation, as well as in the nervous system in *S. frugiperda* [16]. Sensory systems play important roles in insect feeding and identification of host plants [22]. This may be one of the reasons why the invasion ability of *S. frugiperda* is stronger than *S. litura* [16]. In the present study, we found that the upregulated DETs of single-copy orthologous genes in *S. litura* were mainly enriched in energy and metabolic processes, which was opposite to genome-level results. Cui et al., conducted comparative genomic analyses between *S. frugiperda* and *S. litura* that, which identified several *S. frugiperda*-specific positively selected genes related to energy supply [16]. One possible explanation for this result is that we concentrated only on protein-coding genes in the transcriptome analysis, indicating a high proportion of functional genes related to energy for *S. litura*. Among the metabolic mechanisms of resistance that have been discovered, the most common include enhanced insecticide detoxification by upregulated P450, GST and the increased activities of carboxylesterase [32]. By comparing the differences in the aforementioned detoxification pathways between the two species, we found that there existed differences in the expression levels of genes related to detoxification pathways at different stages and species. This provides a theoretical basis for the application of insecticides in practice. For example, the overexpression of P450s is the main cause of pyrethroid resistance, while the contribution of GST and esterase to the resistance is small [36]. We found that the gene expression level involved in the metabolism of xenobiotics by cytochrome P450 pathways in *S. litura* was higher in the larval stage than in chrysalis and adult stages, while that of *S. frugiperda* was highly expressed in all three stages. Therefore, different results may be achieved by using this insecticide in the chrysalis and adult stages of *S. litura* and *S. frugiperda*, which would be affected by their differing resistance to pesticides.

In this study we also investigated the gut microbiome metagenomes of *S. litura* and *S. frugiperda*. *Alphabaculovirus* is an entomopathogenic virus genus [37], and in our results, it was the dominant genus in *S. litura* and *S. frugiperda.* As a lepidopteran-specific nucleopolyhedroviruses, *Alphabaculovirus* could be a candidate genus for RNAi-based pest management strategies [38]. A comparison of the relative abundance of the bacteria from *S. litura* and *S. frugiperda* revealed different microbiota compositions. Firmicutes, Proteobacteria and Actinobacteria were the most dominant phyla in all samples. Firmicutes and Proteobacteria are crucial to maintain insect growth and development during the metabolism of secondary metabolites of host plants [39]. The vast majority of Acinetobacter bacteria have strong drug resistance [40]. Our finding underlined the importance of gut microbiota abundance and composition in the adaptation of insects. At the genus level, the relative abundances of *Clostridium, Glutamicibacter, Streptomyces, Escherichia,* and *Enterobacter* were significantly higher in *S. frugiperda* than in *S. litura*. These bacteria participate in food digestion, nutrition, and the detoxification of plant defense compounds. *Clostridium* has been shown to play a key role in the breakdown of celluloses and insecticide metabolism [41]. *Streptomyce*s can produce antimicrobial secondary metabolites and provide chemical defenses for insects [42]. *Enterobacter* participates in the encoding of carboxylesterase and glutathione S-transferase (GST) and these enzymes are involved in inducing xenobiotic detoxification, which reinforces the supposition that gut microbes can play an important role in insecticide resistance of *S. frugiperda* [43]*.* The relative abundance of *Pediococcus* was significantly higher in *S. litura* than *S. frugiperda*. *Pediococcus* have the ability to decompose organic materials and metabolize several plant secondary biomass to produce phenolic compounds, which are necessary for host insects [39]. Our results demonstrate that these two pests have significantly different gut microbiota genera, among them, the relative abundance of the bacteria involved in detoxification was significantly higher in *S. frugiperda* than *S. litura*.

The *S. litura* and *S. frugiperda* gut metagenome indicated that their gut bacteria may be involved in various metabolic pathways, such as the carbohydrate degradation and detoxification. CAZy database analysis showed that the gut microbes of *S. litura* and *S. frugiperda* were rich in carbohydrate degradation-related genes. GH family enzymes, including β-galactosidase, β-glucosidases, β-glucuronidase, and β-xylosidase, are involved in the utilization of a variety of carbon sources. Pathway analysis revealed that some of the unstratified pathways differed between *S. litura* and *S. frugiperda*. Pentose phosphate pathway and glycogen biosynthesis pathways were more abundant in *S. litura*, which indicates that *S. litura* might have higher production of energy within the gut compared to *S. frugiperda*. In addition, the monothiol biosynthesis pathway was more abundant in *S. frugiperda* and monothiol has antioxidant and detoxification effects [44]. Overall, the data suggest that *S. litura* has more abundant energy production pathways, while the pathway related to detoxification was more abundant in *S. frugiperda*, which is consistent with the findings in our transcriptomics. The difference in energy pathways between the two species may reflect differences in resistance, since insecticide resistance consumes the host’s energy reserves and reduces the energy available for other metabolic functions [45].

*Spodoptera litura* and *S. frugiperda* also differ significantly in terms of ARGs profiles, with *S. frugiperda* having more ARGs than *S. litura*. The most abundant ARG in all samples was DfrA42, encoding dihydrofolate reductase that confers resistance to Trimethoprim. The second most abundant ARG was Efra, heterodimeric ABC transporter efflux pumps that confers resistance to macrolide antibiotic, rifamycin antibiotic and fluoroquinolone antibiotic [46]. Therefore, metagenomic analysis revealed that there were differences at the species and pathway level when comparing *S. litura* and *S. frugiperda*. The energy metabolism related pathway was more abundant in *S. litura* than in *S. frugiperda*. On the contrary, *S. frugiperda* had more abundant gut bacteria associated with detoxification than *S. litura*. The results allowed us to comprehensively analyze the potential functions of gut bacteria contributing to food digestion, nutrition, and metabolic detoxification in *S. litura* and *S. frugiperda*.

It should be mentioned that there are two limitations in this study: (i) Since the adults were collected from the wild, we cannot guarantee the consistency of their rearing environment. (ii) Limitations of only using single-copy orthologous genes to compare the differences between the two pests must be considered. Biological differences may also be influenced by, for example, gene duplications, deletions, and species-specific expansions, which can lead to biological differences between the two species.

## Conclusion

In the present study, the RNA-seq libraries were constructed to investigate the transcript expression patterns during three developmental stages of *S. litura.* A large number of DETs were identified by pairwise comparisons between developmental stages. Several of these DETs were related to detoxification and development. The comparative analysis between *S. litura* and *S. frugiperda* revealed differences in their host adaptation and energy metabolism. We identified the structure of the intestinal microbiota of *S. litura* and *S. frugiperda* based on metagenomics. Our results demonstrated that the relative abundance of gut microbiota involved in detoxification was significantly higher in *S. frugiperda* and the pathway related to detoxification was also more abundant than *S. litura*. Our study provides an important basis for further understanding of the molecular mechanisms of pest development and provides insight into the development of a novel pest control strategy. In the future, more stage-specific pesticide products can be developed through further studies on genes related to detoxification metabolism, which may provide new strategies for effective *S. litura* and *S. frugiperda* control.

## Methods

### Samples collecting

In this study, the adults of *S. litura* and *S. frugiperda* were obtained from cornfields in Xindu District, Chengdu, Sichuan Province, China. They were randomly captured in the cornfields by hand and sweep net. The adults were bred in captivity and artificially propagated to sample their first-generation by the Institution of Plant Protection, Sichuan Academy of Agricultural Sciences. The main food of *S. litura* and *S. frugiperda* was powdery corn. The captive environment was as follows: maintained relative humidity at 70% ± 10% and temperature at 28 °C ± 1 °C in the artificial box, the ratio of light to dark time was 18:6. All samples were the laboratory bred offspring, and the rearing and feeding conditions of captive individuals were consistent.

A total of 15 *S. litura* individuals were used, including three developmental stages: fifth instar larvae (*N* = 3), sixth instar larvae (*N* = 3), chrysalises (*N* = 3), female adults (*N* = 3), and male adults (*N* = 3). According to the developmental stage of *S*. *litura* and consistent with the grouping in *S. frugiperda* study [18], we combined the fifth and sixth instar larvae as the larval group. Larvae removed intact intestinal tissues and the whole bodies from the chrysalis and adult individuals were sampled and mixed with liquid nitrogen, then stored at -80℃. We also sourced the transcriptome data of *S. frugiperda* downloaded from our team’s previous study, which was collected from 15 *S. frugiperda* individuals, with larval stage (fifth instar larvae and sixth instar larvae), chrysalis stage and adult stage (female and male) [18]. The sampling and processing method of *S. frugiperda* is consistent with that of *S. litura.*

### Transcriptome library preparation and sequencing

Trizol reagent (Invitrogen, Carlsbad, CA) was used to extract the total RNA of the samples. The quantity and purity of total RNA were determined by 2% agarose gel electrophoresis. The total RNA purity with OD260/280 was detected by Nanodrop. The RNA density was determined by Qubit and the RNA integrity was analyzed by Agilent 2100. mRNA was collected by Oligo dT enriching beads. First, fragmentation buffer was added to the mRNA to break it into small fragments. Then, the first strand of cDNA was synthesized using random hexamer primers and under the premise of regarding mRNA fragments as a template. Second-strand was synthesized by adding buffer, dNTPs, DNA polymerase I and RNase H. AMPure XP beads was used to purify double-stranded cDNA. After we performed purification, terminal repair, addition of A and ligation of sequencing adaptor, libraries were produced by performing PCR amplification. Finally, the PCR product was purified by AMpure XP beads to obtain the final library. After the library was constructed, Qubit 2.0 was used for preliminary quantification, and the library was diluted to 1.5 ng/mL. Then, Agilent 2100 was used to detect the insert size of the library. After the insert met the expectation, Q-PCR method was used to accurately quantify the effective concentration of the library to ensure library quality. The Illumina Hiseq 2000 platform at Novogene (Novogene, Beijing, China) was used to generate approximately 150 bp paired end (PE) raw reads.

### Transcript data processing

The NGSQC Toolkit version 2.3.3 software [47] was employed to remove adapters and low quality reads (including reads with N bases > 10% and Q-value < 20) to obtain clean reads. Reference genome data and annotation files of *S. litura* were downloaded from the NCBI database (https://www.ncbi.nlm.nih.gov/genome/?term=Spodoptera+litura). The clean reads of each sample were mapped to the reference genome using HISAT2 v2.1.0 software [48]. Samtools v1.9 [49] was then used to convert the output SAM files into BAM files and sort them by chromosome positions. Finally, we obtained a transcript GTF file with the expression information using StringTie v1.3.64 [50]. The raw expression matrix was generated using the prepDE.py script provided by StringTie.

### Functional annotation

Since we lacked functional annotation information in the reference genome of *S. litura*, Gene Ontology (GO) and Kyoto Encyclopedia of Genes and Genomes (KEGG) functional annotations of mRNA sequences were performed. Based on the position annotation from the assembled GTF file of the *S. litura* reference genome, we extracted the mRNA sequences. To obtain comprehensive annotation information of the *S. litura* transcript, the above-mentioned mRNA sequences were aligned to the NCBI (ftp://ftp.ncbi.nlm.nih.gov/blast/db/) nonredundant (NR) protein databases and the Swiss-Prot database using BLASTx with E-value cutoff of 1E-5 [51]. The GO terms information was obtained from the Swiss-Prot results. To obtain a background data set of the KEGG annotation information, nine species (*Drosophila melanogaster, Bombyx Mori, Bombyx mandarina, apilio machaon, Pieris rapae, Danaus plexippus, Helicoverpa armigera, Trichoplusia ni,* and *Plutella xylostella*) were considered as background sets to annotate the best alignment sequences using the KEGG database, which was accomplished by the KEGG Automatic Annotation Server (KAAS) [52]. Further enrichment analysis was performed based on the GO terms and KEGG pathways obtained from the mRNA sequences using ClusterProfiler v3.16.09 [53].

### Differentially expressed transcript analysis and enrichment analysis

The transcripts that had five or more reads in any sample that could be mapped to the reference genome were used for further analysis. All the samples were divided into different groups according to their developmental stages, these being the larval group (hereafter LL, consisting of fifth instar larvae and sixth instar larvae), chrysalis group (i.e., LC), and adult group (i.e., LA, three females and three males). Then, LL versus LC, LC versus LA, and LL versus LA were analyzed using DESeq2 in R packages that used the raw read count matrix as the input (Love et al., 2014). Transcripts in different compared groups with |log twofold change|≥ 2 and *p* values were corrected for multiple testing with the Benjamini–Hochberg false discovery rate (FDR ≤ 0.05) and were considered as DETs.

Transcriptome data of the three developmental stages of *S. frugiperda* were collected from our previous study [18]. There were 15 samples of *S. frugiperda*, with the larval group (hereafter FL, consisting of 5th instar larvae and 6th instar larvae), chrysalis group (i.e., FC), and adult group (i.e., FA, three females and three males). We used the raw read count matrix as the input for subsequent analyses. The OrthoFinder V2.3.117 [54] was used to identify the single-copy orthologous genes between *S. litura* and *S. frugiperda* within the same age stage. GETMM [55] (Gene Length Corrected TMM) method was applied to correct the single-copy orthologous genes that were shared by *S. litura* and *S. frugiperda* in the expression matrix. DETs of the single-copy orthologous genes analyses between LL versus FL, LC versus FC, and LA versus FA were performed by DESeq2 after the expression matrix was normalized, and we set the FDR ≤ 0.05 and | log 2 FC|≥ 2 as the standard to select the DETs between the two species. Functional enrichment analyses of DETs between and within developmental stages and between *S. litura* and *S. frugiperda* were performed using the GO term and KEGG pathway background data sets constructed according to *S. litura* mRNA sequences. In our study, the *p* values were adjusted by the BH method, and FDR ≤ 0.05 was considered as significantly enriched GO terms and KEGG pathways by ClusterProfiler v3.16.0 [53].

### Metagenomic sequencing and profiling

The larval samples of *S. litura* and *S. frugiperda* were the laboratory bred offspring of wild-caught individuals, consequently environment and diet were consistent. We collected 12 fecal samples from *S. litura* and *S. frugiperda* (fifth and sixth instar larvae) and extracted 0.2 μg DNA per sample that was used as input material for the DNA library preparations. Genomic DNA was tested for quality, with quality genomic DNA samples being fragmented by sonication to a size of 350 bp. DNA fragments were then endpolished, A-tailed, and ligated with the full-length adapter for Illumina sequencing, followed by further PCR amplification. After PCR products were purified by AMPure XP system (Beckman Coulter, Beverly, USA), DNA concentration was measured by Qubit®3.0 Flurometer (Invitrogen, USA), libraries were analyzed for size distribution by NGS3K/Caliper and quantified by real-time PCR (3 nM). The DNA libraries were then sequenced on Illumina platform and paired-end reads were generated.

Quality control and trimming of sequences were conducted by KneadData (version 0.7.4) toolkit. Contamination sequences of the host *S. litura* and *S. frugiperda* genome were removed by KneadData integrated Bowtie2 tool (version 2.3.4.1) [56]. The metagenomic data were assembled with MEGAHIT (version 1.2.9) [57]. Taxonomic classification was performed by standard taxonomic sequence classification tools, Kraken2 (version 2.1.1) [58]. Contigs were predicted for open reading frames (ORFs) via Prodigal (version 2.6.3) [59]. Cd-Hit (version 4.8.1) [60] was applied to build non-redundant gene sets for all predicted genes with more than 95% identity and more than 90% coverage. The gene with the longest full length from each cluster was selected as the representative read of each gene set. The abundance information of each gene in each sample was calculated by Salmon (version 1.5.2) [61]. To obtain the information about carbohydrate active enzymes, sequences were compared in the Carbohydrate-Active enzymes (CAZy) databases using dbCAN2 [62]. Resistance Gene Identifier (RGI) in the Comprehensive Antibiotic Resistance Database (CARD) was used to predict resistant gene from protein data [63]. The metabolic functional profile was estimated using HUMAnN3 with the full UniRef90 database [64]. Linear discriminant analysis (LDA) effect size (LEfSe) was performed to identify differentially abundant species and pathways between groups.

## Supplementary Information

**Additional file 1.** Supplementary Figures

**Additional file 2.** Supplementary Tables  

## Data Availability

The raw sequencing reads from this study have been submitted to the CNGBdb with the project accession CNP0001314 (available at https://db.cngb.org/search/project/CNP0001314/).
