# Supplementary material for: Transcriptomics and metagenomics of common cutworm (Spodoptera litura) and fall armyworm (Spodoptera frugiperda) demonstrate differences in detoxification and development
Source: BMC Genomics. 2022 May 20;23:388. doi: 10.1186/s12864-022-08613-6 (PMC9123734; doi:10.1186/s12864-022-08613-6)
Supplement: Supplementary file 1 — Additional file 1. Supplementary Figures [file 12864_2022_8613_MOESM1_ESM.docx]

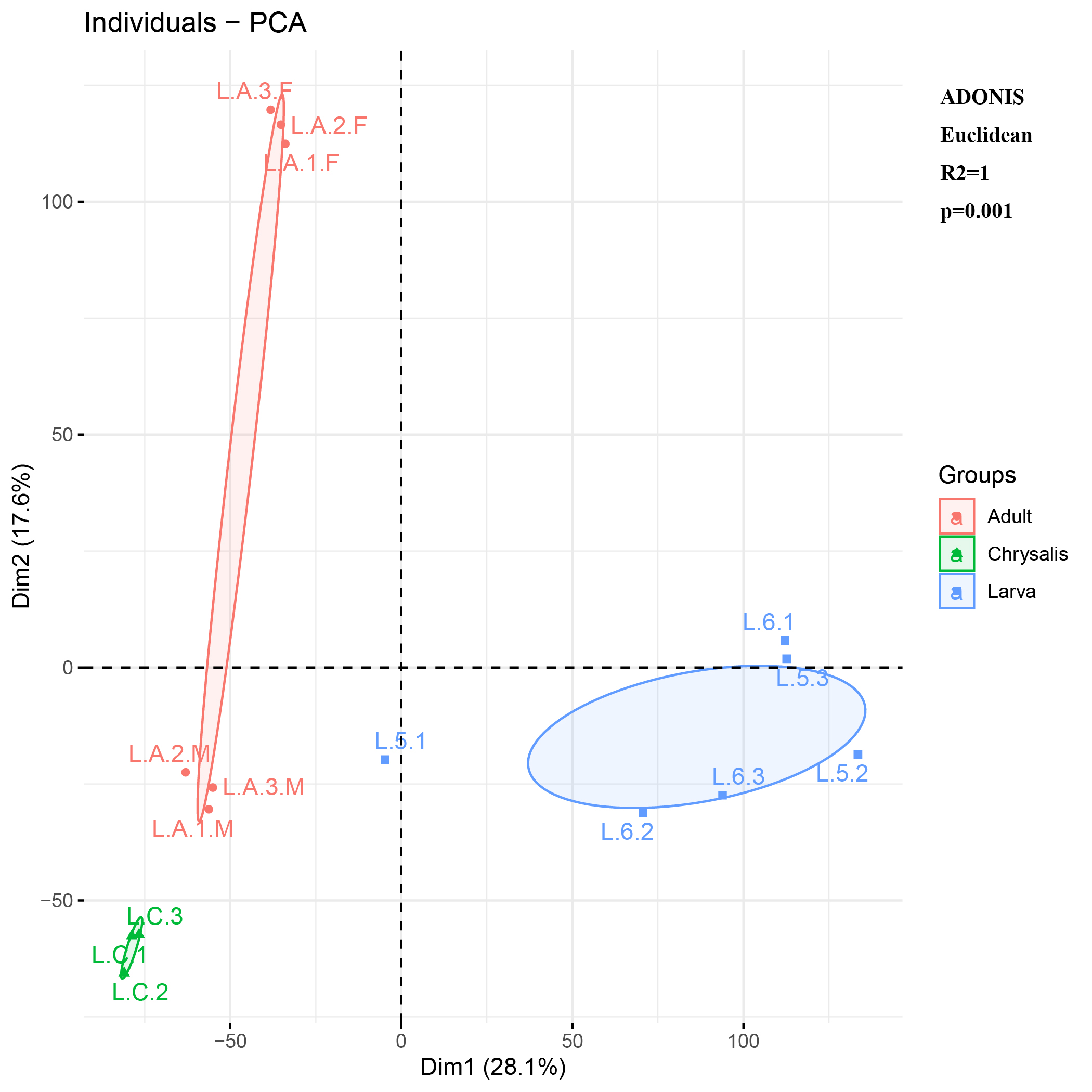


**FIG. S1.** The principal components analysis of three developmental stages in S. litura. The expression of transcripts was transformed using rlog function.


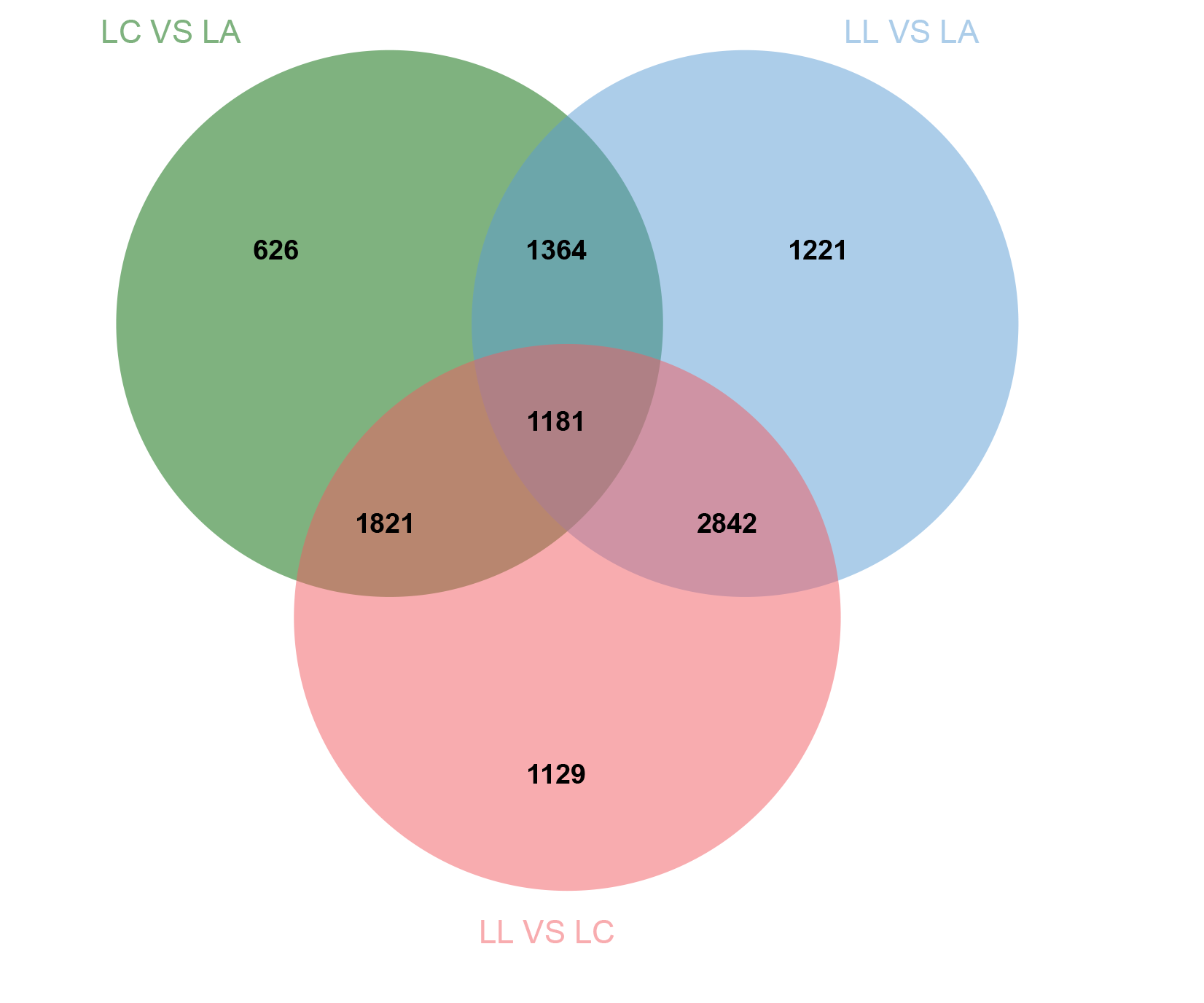


**FIG. S2.** The Venn diagram of DETs between pairwise comparison groups of three developmental stages in *S. litura*.


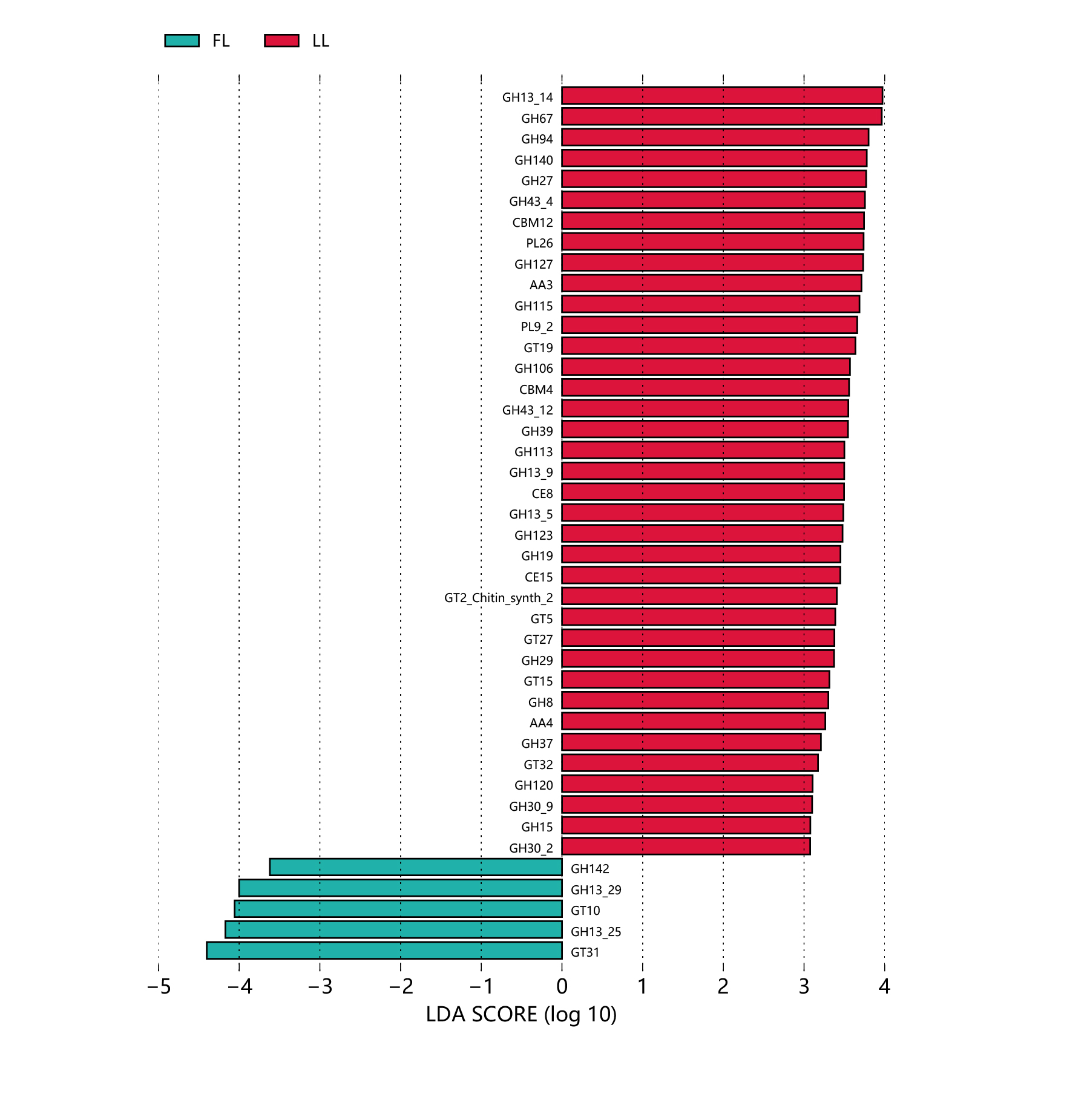


**FIG. S3.** LDA effect size (LEfSe) analysis of the function of Carbohydrate-Active enzymes (CAZy) between the two groups.
